# Gut Microbiota‐Derived Butyrate Strengthens Blood‐Brain Barrier Integrity and Attenuates *Streptococcus suis SC19*‐Induced Meningitis: Involvement of the Gut‐Brain Axis

**DOI:** 10.1155/tbed/5502623

**Published:** 2026-08-18

**Authors:** Wangyuan Yao, Meili Chen, Huachun Pan, Md. F. Kulyar, Jialu Zhang, Hongkai Ren, Qingqing Luo, Hongdou Liu, Qinghua Wang, Xuyuan Wang, Rendong Fang

**Affiliations:** ^1^ College of Veterinary Medicine, Southwest University, Chongqing 400010, China, swu.edu.cn; ^2^ College of Life Sciences, Longyan University, Longyan 364000, China, lyun.edu.cn; ^3^ Department of Regenerative Medicine, State Research Institute Centre for Innovative Medicine, LT-08406 Vilnius, Vilnius, Lithuania, imi.lt; ^4^ College of Animal Science and Technology, Ningxia University, Yinchuan 750021, China, nxu.edu.cn

**Keywords:** blood-brain barrier, butyrate, gut-brain axis, Nrf2/NF-κB, S*treptococcus suis* serotype 2, short-chain fatty acids

## Abstract

*Streptococcus suis* serotype 2 (*SS2*) is a major zoonotic pathogen that causes severe meningitis and high mortality in both humans and swine. Growing evidence shows that metabolites generated along the gut‐brain axis regulate neuroinflammation. In this study, we demonstrate that oral administration of sodium butyrate (NaB) (300 or 600 mg/kg/day) for 28 days lowered mortality and lessened neuropathological signs in mice infected with *SS2* (*SC19* strain). Butyrate prophylaxis was associated with lower bacterial loads in the blood and brain tissue, preserved expression of tight junction proteins (ZO‐1, Claudin‐5, and Occludin) in the brain, and maintained blood‐brain barrier (BBB) integrity and permeability. 16S rRNA sequencing analysis revealed that *SC19* infection significantly diminished the diversity and richness of the gut microbiota, notably depleting key short‐chain fatty acid (SCFA)‐producing bacterial taxa. Butyrate intervention restored microbial homeostasis and enriched SCFA‐producing communities. Mechanistically, butyrate was associated with upregulated expression of nuclear factor erythroid 2‐related factor 2 (Nrf2) and its downstream antioxidant effectors (heme oxygenase 1 [HO‐1] and NQO1), while inhibiting phosphorylation of NF‐κB p65 and the secretion of proinflammatory cytokines (interleukin‐1β [IL‐1β], interleukin‐6 [IL‐6], and tumor necrosis factor‐α [TNF‐α]) in human cortical microvessels endothelial cells/D3 (hCMEC/D3) cells. Critically, these protective effects were abrogated upon pharmacological inhibition of Nrf2, indicating the critical role of Nrf2 in butyrate‐mediated attenuation of neuroinflammation and oxidative stress. Our findings highlight the prophylactic potential of modulating the gut‐brain axis as a novel strategy to mitigate central nervous system (CNS) infections associated with foodborne pathogens.

## 1. Introduction

*Streptococcus suis* serotype 2 (*SS2*) is a major Gram‐positive zoonotic pathogen that causes substantial economic losses in the global swine industry and represents a serious public health concern, particularly as a leading etiological agent of bacterial meningitis in humans [1]. Human infections, which predominantly affect individuals with occupational exposure to pigs or pork‐derived products, often result in life‐threatening conditions such as meningitis, septic shock, and streptococcal toxic shock‐like syndrome (STSLS) [2]. The neuropathogenesis of *SS2* is intimately associated with its capacity to traverse the blood‐brain barrier (BBB), a highly selective endothelial structure that protects the central nervous system (CNS) [3]. *SS2* employs an array of virulence factors, including suilysin, the capsular polysaccharide, fibronectin‐binding proteins, and enolase, to adhere to and invade brain microvascular endothelial cells, thereby disrupting tight‐junction proteins (ZO‐1, Claudin‐5, and Occludin) and increasing paracellular permeability [4–6]. This disruption is accompanied by intense neuroinflammatory responses mediated by the release of proinflammatory cytokines and matrix metalloproteinases (MMPs), which further exacerbate BBB damage and neuronal injury [7]. Although significant progress has been made in elucidating the pathogenic mechanisms of *SS2*, current research remains constrained by a predominant focus on either bacterial virulence determinants or generalized enhancement of host immune surveillance. Nevertheless, the prevention and control of *SS2* continue to pose considerable challenges worldwide. While antibiotics are effective in reducing the bacterial burden in tissues, they fail to halt the rapid compromise of the BBB exacerbated by inflammatory processes [8]. Moreover, the growing threat of antibiotic resistance presents a substantial and often irreversible constraint on treatment efficacy. Thus, there is an urgent need to develop host‐directed preventive strategies that can complement conventional antibiotics and improve clinical outcomes. Research focused on preserving BBB integrity and modulating neuroinflammation represents a promising frontier for alleviating the severe consequences of *SS2* infection.

The gut‐brain axis, a sophisticated bidirectional communication network integrating neurological, immunological, and endocrine signaling between the gastrointestinal tract and the CNS, has emerged as a critical regulator of CNS homeostasis [9]. Central to this axis are the gut microbiota and their metabolites, which influence neuroinflammation, BBB integrity, and neuronal function [10, 11]. Dysbiosis of the gut microbiome has been mechanistically linked to numerous neurological disorders through aberrant immune activation and impaired barrier function [12]. For instance, in Alzheimer’s disease, microbiota‐derived amyloids can cross‐seed cerebral Aβ aggregation via peripheral inflammatory priming [13], while in Parkinson’s disease, gut‐derived α‐synuclein fibrils propagate via the vagus nerve, triggering neuroinflammation and Lewy body formation [14]. Recent multiomics studies further reveal that gut microbes regulate microglial maturation and synaptic pruning through complement cascades and short‐chain fatty acid (SCFA) signaling, thereby influencing cognitive outcomes [15]. In ischemic stroke models, microbiota‐modulated IL‐17^+^ γδ T cells migrate to the meninges, exacerbating neuronal injury through cytokine release [16]. These findings underscore the gut‐brain axis as an integrated physiological system whose dysregulation contributes fundamentally to CNS disease pathogenesis, highlighting its potential as a target for therapeutic intervention.

Among gut microbiota‐derived metabolites, SCFAs, particularly butyrate, serve as pivotal mediators of host‐microbe crosstalk with profound implications for CNS health [17]. Butyrate is primarily generated in the colon through microbial fermentation of resistant starch and dietary fibers by commensals such as *Faecalibacterium prausnitzii* and *Roseburia* species [18]. Beyond its role as a colonocyte fuel, butyrate acts as a histone deacetylase (HDAC) inhibitor, thereby remodeling chromatin and reprogramming genes that control inflammation, oxidative stress, and barrier function [19, 20]. Mechanistic studies elucidate that butyrate enhances intestinal tight junction assembly and concurrently strengthens the BBB by preserving endothelial junctional complexes and reducing MMP‐9 activity [21, 22]. Butyrate also signals through G‐protein‐coupled receptors (GPR41, GPR43, and GPR109a) expressed on immune and neural cells, modulating neuroimmune responses. These multifaceted mechanisms, spanning epigenomic regulation, immunometabolism, and barrier stabilization, collectively underscore the therapeutic potential of butyrate in counteracting neuroinflammation, proteopathy, and neuronal dysfunction across diverse neurological conditions. However, despite accumulating evidence for the neuroprotective effects of butyrate in sterile injury models such as stroke, traumatic brain injury, and neurodegenerative proteinopathies, systematic validation against bacterial CNS pathogens remains surprisingly limited. Specifically, it is unknown whether oral butyrate administration can protect against highly virulent *SS2*‐induced meningitis by modulating the gut‐brain axis.

Thus, 6–8‐week‐old C57BL/6 female mice were used to establish the murine model using *SS2* (strain *SC19*). This study aimed to evaluate whether butyrate intervention alleviates meningitis severity and associated symptoms, modulates gut microbial diversity, and enhances the abundance of beneficial bacteria. Furthermore, using human cortical microvessels endothelial cells/D3 (hCMEC/D3) cells, we elucidated the molecular mechanisms by which butyrate preserves BBB integrity, focusing on the nuclear factor erythroid 2‐related factor 2 (Nrf2)/NF‐κB pathway. We therefore hypothesized that butyrate protects against *SS2* induced meningitis through the gut brain axis by restoring microbial homeostasis, elevating systemic and cerebral SCFAs, and suppressing neuroinflammation via Nrf2 dependent mechanisms.

## 2. Materials and Methods

### 2.1. Reagents

Sodium butyrate (NaB) (Lot: F17HS175370, reagent grade, 98%) was purchased from Shanghai Yuanye Biotechnology Co., Ltd. Evans Blue (Lot: 33724025ZT, 2% EB) staining solution and 4% paraformaldehyde were purchased from Biosharp (Lot: 25541786, Beijing, China). Todd‐Hewitt Broth (THB) medium was purchased from Hopebio (Lot: HB0311‐3, Qingdao, China). The sterility of each lot was confirmed by incubation for 48 h before use. Enzyme‐linked immunosorbent assay (ELISA) kits for interleukin‐1β [IL‐1β] (Lot: BMS6002‐2), interleukin‐6 [IL‐6] (Lot: BMS603‐2), and tumor necrosis factor‐α [TNF‐α] (Lot: 88‐7324‐88) were purchased from Thermo Scientific (Massachusetts, USA). Brusatol was purchased from Abmole (Lot: M8710, Shanghai, China). RIPA Lysis Buffer was purchased from Solarbio (Lot: 2500120013, Beijing, China). Primary antibodies of Occludin (A2601, 1:1500 for WB), Claudin‐5 (A10207, 1:1500 for WB), and phospho‐NF‐κB p65/RelA‐S536 (AP0475, 1:20000 for WB) were purchased from ABclonal (Wuhan, China). The antibody of ZO‐1 (D364329, 1:1000 for WB) was purchased from BBI (Shanghai, China). The antibodies of NRF2 (WL02135, 1:1000 for WB), HO‐1/heme oxygenase 1 (WL02400, 1:1000 for WB), and NQO1 (WL04860, 1:1000 for WB) were purchased from Wanleibio (Shenyang, China). The antibody of GAPDH (10494‐1‐AP, 1:10000 for WB) and horseradish peroxidase (HRP)‐conjugated goat anti‐rabbit IgG (SA00001‐2, 1:10000 for WB) were purchased from Proteintech (Wuhan, China).

### 2.2. Experimental Animals, Bacteria, and Cell Lines

Female C57BL/6J SPF mice (aged 6 weeks and weighing 18–22 g) were obtained from Hunan SJA Laboratory Animal Co., Ltd. Sentinel screening confirmed the absence of *Helicobacter* spp. and mouse norovirus. Before experimentation, mice (10 mice per cage) were placed in a standard environment (temperature 26 ± 2°C, humidity 55% ± 5%, and light/dark cycle of 12 h) for 1 week and were fed ad libitum. All animal procedures were approved by the Institutional Animal Care and Use Committee (IACUC) of Southwest University, Chongqing, China (IACUC‐20221022‐02). Environmental enrichment (nestlets and shelters) was provided throughout.

The highly virulent *SC19* strain of *SS2* was kindly provided by Professor Xiangru Wang’s lab at Huazhong Agricultural University. *SC19* was inoculated into THB containing 5% fetal bovine serum (FBS) and incubated at 37°C with orbital shaking at 200 rpm. The hCMEC/D3 cell line was purchased from Jenniobio Biotechnology Co., Ltd. (Guangzhou, China). Cells were maintained in complete EGM‐2 medium (Lonza, Basel, Switzerland) supplemented with 10% FBS (ZETA, Australia origin, USA) and cultured at 37°C in a 5% CO_2_ incubator.

### 2.3. Study Design

#### 2.3.1. In Vivo Study

**Figure 1:**
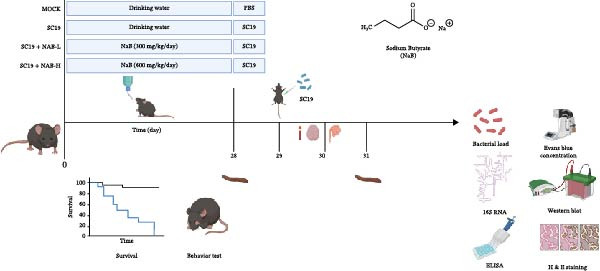
Schematic overview of the experimental strategy.

After 1 week of acclimation, all mice were randomly assigned into five groups (*n* = 16/group), as summarized in Figure 1: (1) Mock group: free access to drinking water, intraperitoneally injected with phosphate‐buffered saline (PBS) after 4 weeks; (2) *SC19* group: free access to drinking water, intraperitoneally injected with logarithmic‐phase *SC19* bacterial culture after 4 weeks; (3) *SC19* + NaB‐L group: orally gavaged with NaB dissolved in drinking water at a final dose of 300 mg/kg/day, followed by intraperitoneal injection of logarithmic‐phase *SC19* bacterial culture after 4 weeks; (4) *SC19* + NaB‐H group: orally gavaged with NaB dissolved in drinking water at a final dose of 600 mg/kg/day, followed by intraperitoneal injection of logarithmic‐phase *SC19* bacterial culture after 4 weeks; (5) NaB group: mice receiving 600 mg/kg/day NaB without *SC19* infection. The NaB doses were based on a published study and have been confirmed in our preliminary experiments.

For the survival study, following 28 days of NaB administration, mice were challenged intraperitoneally with *SC19* (1.5 × 10^8^ colony‐forming units [CFU] per mouse). The actual CFU was verified by retrospective plating in triplicate. Mortality was subsequently monitored and recorded every 6 h for each experimental group. Humane endpoints (≥20% weight loss or severe neurological deficit) were predefined. For meningitis establishment, mice were challenged intraperitoneally with *SC19* (1 × 10^8^ CFU per mouse). Existing studies combined with preliminary experiments have established 1.5 × 10^8^ CFU as the minimum lethal dose, which is therefore employed in survival studies. Conversely, a dose of 1 × 10^8^ CFU is nonlethal but reliably elicits meningitis symptoms, rendering it the optimal dose for modeling meningitis. Approximately 48 h postinfection, mice began to exhibit clinical neurological signs consistent with bacterial meningitis; the meningitis model success rate exceeded 50%. Mice were euthanized at 48 h postinfection. Only mice that successfully developed meningitis at this time point were included in the final analysis, with an equal number of mice per group.

#### 2.3.2. In Vitro Study

Before experimental treatments, hCMEC/D3 cells in good condition were passaged from culture flasks into 12‐well plates and allowed to adhere for 12 h. *SS2* (*SC19*) was cultured until the OD_600_ reached 0.5–0.6, after which the bacterial suspension was temporarily stored at 4°C. An aliquot corresponding to the desired multiplicity of infection (MOI) was centrifuged at 3000 rpm for 10 min, the supernatant was discarded, and the pellet was resuspended in EGM‐2 medium.

hCMEC/D3 cells were assigned to four experimental groups (with three biological replicates per group): (1) Mock group: Cells were maintained in a normal culture medium. (2) *SC19* group: after 40 h of culture, cells were infected with *SC19* (MOI = 10) for 8 h. (3) *SC19* + NaB‐L group: cells were treated with 0.5 mM NaB for 40 h (the 40 h pretreatment duration for NaB was determined based on time‐course pilot experiments showing maximal cytoprotective effects without cytotoxicity), followed by infection with *SC19* (MOI = 10) for 8 h. (4) *SC19* + NaB‐H group: cells were treated with 1 mM NaB for 40 h, followed by infection with *SC19* (MOI = 10) for 8 h. Cell monolayers were ~90% confluent at the time of infection to mimic a mature endothelium.

For the Nrf2 inhibition assay, following 40 h of NaB treatment, as outlined above, cells were washed twice with PBS. All NaB‐treated groups were then incubated with culture medium containing 50 nM Brusatol (Nrf2 inhibitor, 1 mg Brusatol dissolved in 0.19 mL of DMSO to form a 10 mM mother solution and then diluted with cell culture medium to form a 50 nM working solution) for 18 h, followed by infection with *SC19* (MOI = 10) for 8 h. An inhibitor control group was included: cells were treated with culture medium containing 50 nM Brusatol for 18 h without NaB pretreatment, followed by infection with *SC19* (MOI = 10) for 8 h. The doses of NaB and Brusatol are based on a published study and were confirmed in our preliminary experiments.

### 2.4. Bacterial Load Assessment

Upon the manifestation of neurological symptoms indicative of successful meningitis induction, bacterial loads in the brain, intestinal tissue (cecum and colon), and blood of mice were quantified. Tissue separated in a sterile environment was homogenized in sterile PBS, and serial dilutions of the homogenates and blood were plated onto THB agar plates. After incubation at 37°C for 12–15 h, bacterial colonies were enumerated. Only plates containing 30–300 colonies were considered. Counts outside this range were excluded to maintain quantitative accuracy. Results are expressed as CFU per gram of tissue or per milliliter of blood.

### 2.5. Evans Blue Staining for BBB Integrity

Following successful induction of meningitis, mice from each group were administered 100 μL of 5% Evans blue dye via tail vein injection. Twenty‐four hours postinjection, the mice were anesthetized and transcardially perfused with physiological saline. Subsequently, the mice were euthanized, and intact brain tissues were carefully collected. After gently blotting excess blood with filter paper, the brains were photographed against a white background to assess Evans blue extravasation. For quantitative analysis, a portion of each brain tissue was placed in a preweighed 2 mL microcentrifuge tube containing grinding beads, and the exact weight was recorded. Then, 500 μL of formamide was added to each tube, and the samples were homogenized to facilitate complete dye extraction. The homogenates were incubated at 50°C for 48 h and then centrifuged at 1000 rpm for 10 min. The supernatant was collected, and the Evans blue content was quantified spectrophotometrically by measuring absorbance at 620 nm. The measured absorbance was normalized to the tissue wet weight (OD_620_ nm per gram of brain tissue). All image acquisition and quantitative absorbance measurements were performed by an investigator blinded to the experimental group assignments.

### 2.6. Quantification of Inflammatory Factors via ELISA

Following euthanasia, blood samples were collected from mice via retro‐orbital bleeding. The blood was allowed to clot at room temperature for 30 min and then centrifuged at 2000 rpm for 15 min at 4°C to obtain the serum. The supernatant (serum) was aliquoted and stored at −80°C until further analysis. For brain tissue homogenates, brain samples were homogenized in ice‐cold PBS containing a protease inhibitor cocktail. The homogenates were centrifuged at 12,000 rpm for 15 min at 4°C. The resulting supernatants were collected, aliquoted, and stored at −80°C. The culture medium from hCMEC/D3 cells was collected and centrifuged at 1000 rpm for 10 min to remove cellular debris. The clarified supernatants were aliquoted and stored at –80°C before the assay. Freeze‐thaw stability was verified for all cytokines to ensure measurement fidelity.

The concentrations of IL‐1β, IL‐6, and TNF‐α in serum, brain homogenates, and cell culture supernatants were quantified using commercial ELISA kits according to the manufacturer’s instructions. Briefly, 100 μL of each standard and prepared sample was added to the appropriate wells of the antibody‐precoated plate and incubated for the specified duration. After incubation and washing to remove unbound materials, a biotin‐conjugated detection antibody was added to each well, followed by incubation with a streptavidin‐HRP conjugate. A tetramethylbenzidine (TMB) substrate solution was then added to develop color. The enzyme reaction was stopped by adding a stop solution, and the optical density (OD) of each well was immediately measured at 450 nm using a microplate reader. The final cytokine concentrations in brain tissue homogenates were normalized to total protein content, as determined by a bicinchoninic acid (BCA) protein assay, and expressed as picograms per milligram of protein (pg/mg). The concentrations in serum and cell culture media are expressed as picograms per milliliter (pg/mL).

### 2.7. Evaluation of Neurobehavioral Symptoms

Referring to a published study [23] and confirmed in our preliminary experiments, we established the four‐point clinical Neurocore scale for mice as follows: 0 points: normal behavior and posture without obvious abnormalities; 1 point: sluggish movement and hunched back; 2 points: crouching immobility and exophthalmos; 3 points: head and neck twitching with body tremors; and 4 points: head and neck bending and opisthotonus. Five staff members not involved in this study were randomly selected to independently observe and score the mice within a fixed time period. Interobserver reliability (*κ* = 0.87) confirmed the consistency of behavioral scoring.

### 2.8. Histological Analysis of Brain and Colon Tissues

At the designated experimental endpoints, the mice were humanely euthanized. For brain tissue collection, transcardiac perfusion was performed. Briefly, the circulatory system was first flushed with ice‐cold PBS (Servicebio, China) to remove blood, followed by perfusion with 4% paraformaldehyde (Servicebio, China) in PBS for fixation. The entire brain was then carefully dissected and postfixed in 4% paraformaldehyde at 4°C for 24 h. For colon tissue collection, the cecum was promptly isolated without prior perfusion. Representative segments were collected and fixed in 4% paraformaldehyde at room temperature for 48 h.

Following fixation, all tissue samples were dehydrated through a graded ethanol series (70%, 80%, 90%, and 95%), cleared in xylene, and embedded in paraffin wax. Serial sections of 4–5 µm thickness were obtained using a microtome. For histological analysis, the tissue sections were deparaffinized, rehydrated, stained with hematoxylin and eosin (H&E) and periodic acid‐Schiff (PAS) stain (Solarbio, China) following standard protocols. In brief, sections were stained with hematoxylin to visualize nuclei, differentiated, and blued, and subsequently counterstained with eosin to delineate cytoplasmic and extracellular matrix components. This procedure ensures high‐quality tissue preservation and staining, enabling detailed histological evaluation of the tissue morphology and structural integrity. Adjacent sections were reserved for immunohistochemistry to correlate the structure with molecular changes.

### 2.9. Western Blotting Analysis

Brain tissue samples were thoroughly homogenized in RIPA lysis buffer (Solarbio, China) with 1 mM phenylmethylsulfonyl fluoride (PMSF) (Solarbio, China). hCMEC‐D3 cell samples were lysed in RIPA containing PMSF for 30 min on ice. According to the manufacturer’s instructions, protein concentrations were quantified using a BCA assay kit (Beyotime, China). Protein samples were mixed with 5× loading buffer (Solarbio, China), denatured at 100°C for 10 min, and separated by SDS‐PAGE (8%–12% gels) with equal protein loading. Subsequently, proteins were electrophoretically transferred onto a PVDF membrane using a wet‐transfer system at 250 mA for 2 h on ice. The membrane was blocked with 5% nonfat milk in TBST for 1 h at room temperature, followed by overnight incubation at 4°C with specific primary antibodies diluted in TBST (details are provided in the reagents). After washing, the membrane was incubated with an HRP‐conjugated secondary antibody for 1 h at room temperature. Protein bands were visualized using an enhanced chemiluminescence (ECL) substrate (Meilunbio, China) and imaged with a chemiluminescence detection system. Band intensities were quantified by densitometry using Image Lab software (Bio‐Rad, USA) and normalized to GAPDH. Calibration curves with recombinant standards confirmed linear signal detection. Densitometric values were normalized to a pooled internal control loaded on every gel. Biological replicates: All Western blot experiments were performed using three biological replicates (*n* = 3 per group for animal tissues and three independent cell passages for in vitro experiments). Representative blots from one experiment are shown; densitometric quantifications represent the mean ± standard deviation (SD) of all replicates. Normalization: confirm through the BCA assay before electrophoresis that the protein concentration of the loading sample has been adjusted to be consistent. GAPDH was used as the loading control.

### 2.10. SCFAs (Acetate, Propionate, and Butyrate) Levels Analysis

For brain tissue analysis, ~100 mg of the sample was homogenized with 500 μL of extraction solution (methanol:water = 4:1) using a cryogenic grinder (6 min, −10°C, 50 Hz). The homogenate was sonicated (30 min, 5°C, 40 kHz), incubated at −20°C for 30 min, and centrifuged (15 min, 4°C, 13,000 rpm). A 20 μL aliquot of the supernatant was derivatized with 20 μL of 200 mM 3NPH·HCl and 20 μL of 120 mM EDC·HCl (containing 6% pyridine) at 40°C for 30 min. The mixture was then diluted to 1000 μL with 50% acetonitrile in water before liquid chromatography‐mass spectrometry (LC‐MS)/MS analysis. For serum samples, 50 μL of serum was mixed with 100 μL of acetonitrile (Fisher, USA), sonicated under the same conditions, and then centrifuged. The supernatant was derivatized identically and diluted to 750 μL with 50% acetonitrile. Chromatographic separation was performed on a BEH C18 column (150 × 2.1 mm^2^, 1.7 μm) using a gradient of 0.1% formic acid in water (A) and 0.1% formic acid (Fisher, USA) in acetonitrile (B) at 0.35 mL/min and 40°C. The gradient program was as follows: 10% B (0–2 min), increased to 55% B (2–11 min), to 95% B (11–12 min), held at 95% B (12–13 min), returned to 10% B (13–13.1 min), and re‐equilibrated at 10% B (13.1–16 min). Mass spectrometric detection was conducted in negative‐ion mode with the following parameters: curtain gas at 35 psi, collision gas medium, ion spray voltage at −4500 V, temperature at 450°C, and ion source gas 1 and gas 2 at 40 psi each. Data were processed using AB Sciex OS software. Peak integration was performed automatically with manual verification. Quantification was based on external calibration curves constructed by plotting the peak area against analyte concentration.

### 2.11. Molecular Docking

The three‐dimensional (3D) structures of butyrate and the Nrf2 gene were retrieved from the PubChem database and the Protein Data Bank (PDB, AF_AFQ60795F1), respectively. Molecular docking was performed using AutoDock Vina, and binding affinity values were used to evaluate the docking results. The interaction profiles were subsequently visualized in PyMOL.

### 2.12. 16S rRNA Sequencing of Gut Microbial Diversity Analysis

Following euthanasia, the colon was aseptically dissected, and its contents were collected into a sterile bag, which was immediately sealed and flash‐frozen on dry ice. For 16S rRNA sequencing analysis, six mice were randomly selected from each experimental group (*n* = 6/group). Samples were transferred to the laboratory and stored at –80°C until DNA extraction. Total genomic DNA was extracted from cecal contents using the E.Z.N.A. Soil DNA Kit (Omega Bio‐tek, Norcross, GA, USA) following the manufacturer’s protocol. DNA quality and concentration were assessed by 1.0% agarose gel electrophoresis and a NanoDrop 2000 spectrophotometer (Thermo Scientific, USA), respectively. DNA aliquots were stored at –80°C before downstream analysis. The hypervariable V3–V4 region of the bacterial 16S rRNA gene was amplified with primers 338F (5^′^‐ACTCCTACGGGAGGCAGCAG‐3^′^) and 806R (5^′^‐GGACTACHVGGGTWTCTAAT‐3^′^) using a T100 Thermal Cycler (Bio‐Rad, USA). Each 20 µL PCR reaction contained 4 µL of 5× Fast Pfu buffer, 2 µL of 2.5 mM dNTPs, 0.8 µL of each primer (5 µM), 0.4 µL of Fast Pfu polymerase, 10 ng of template DNA, and nuclease‐free water to volume. The PCR conditions were as follows: 95°C for 3 min; 27 cycles of 95°C for 30 s, 55°C for 30 s, and 72°C for 45 s, followed by a final extension at 72°C for 10 min. To control for contamination, negative controls (blank swabs and no‐template PCR controls) were processed in parallel; no bacterial DNA was detected in any negative control. PCR products were separated on a 2% agarose gel, purified using a PCR Clean‐Up Kit (YuHua, Shanghai, China), and quantified with a Qubit 4.0 fluorometer (Thermo Fisher Scientific, USA). Purified amplicons were pooled in equimolar ratios and subjected to paired‐end sequencing (2 × 250 bp) on an Illumina NovaSeq 6000 platform (Illumina, San Diego, CA, USA) by Majorbio Bio‐Pharm Technology Co., Ltd. (Shanghai, China), following standard protocols. Raw sequencing data were demultiplexed and quality‐controlled using fastp (v0.19.6) to remove low‐quality reads. Paired‐end reads were merged using FLASH (v1.2.11). High‐quality sequences were denoised into amplicon sequence variants (ASVs) using the DADA2 plugin within QIIME 2 (version 2024.2), which models and corrects sequencing errors to resolve true biological sequences. To account for uneven sequencing depth, samples were rarefied to 20,000 sequences per sample for downstream diversity analyses, which retained an average Good’s coverage of 97.90%. Beta‐diversity metrics were center‐log‐ratio‐transformed to address compositionality. This transformation allowed direct comparison of the community structure across conditions without bias from sparsity. Taxonomic classification of ASVs was performed using a naïve Bayes classifier trained on the SILVA 132 database. The functional potential of microbial communities was predicted using PICRUSt2 (Phylogenetic Investigation of Communities by Reconstruction of Unobserved States), which infers gene families and metabolic pathways from ASV phylogenetic placement. All analyses were conducted on the Majorbio Cloud Platform (https://cloud.majorbio.com).

### 2.13. Statistical Analysis

All experiments were conducted with at least three independent replicates to ensure robustness and reproducibility. Data are expressed as the mean ± SD. Shapiro–Wilk and Levene’s tests confirmed the normality and homogeneity of variance. Statistical analyses were performed using GraphPad Prism 7 software (GraphPad Software, USA). Differences between experimental groups were evaluated using Student’s *t*‐test for pairwise comparisons or one‐way analysis of variance (ANOVA) followed by Dunnett’s multiple‐comparison test for multigroup comparisons. Survival curves were analyzed using the log‐rank test. Neurobehavioral scores were analyzed using the Kruskal–Wallis test, followed by Dunn’s multiple comparisons test. For microbiome analysis, alpha diversity was compared by Kruskal–Wallis test, beta diversity was evaluated based on Bray–Curtis dissimilarity using principal coordinate analysis (PCoA) in R. Statistical significance of beta diversity was assessed using permutational multivariate analysis of variance (PERMANOVA), and differential genus abundance by Wilcoxon rank‐sum test with Benjamini–Hochberg FDR correction. LEfSe identified taxa with LDA. Correlations were evaluated using Spearman’s rank test.

## 3. Results

### 3.1. Butyrate Intervention Effectively Ameliorated Meningitis Induced by *Streptococcus suis SC19* in Mice

**Figure 2:**
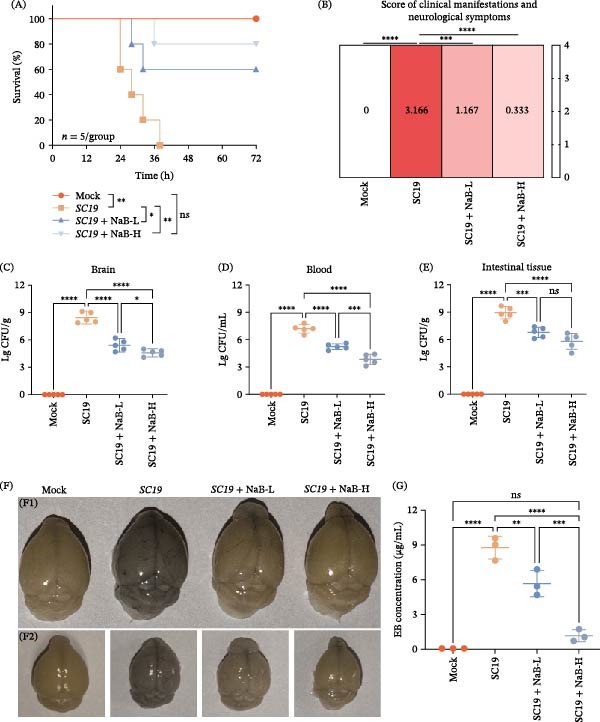
Effects of butyrate administration on the survival rate, clinical symptoms, bacterial load, and blood‐brain barrier of *SC19*‐infected mice. (A) Survival status of three groups of mice infected with *SC19* after intraperitoneal injection, with the mock group receiving simulated intraperitoneal infection. Curves were analyzed using the log‐rank test (*n* = 5). (B) The clinical neurological symptom scores of mice in each group after *SC19* infection, with detailed scoring criteria provided in Section 2 (*n* = 5). Load of *Streptococcus suis SC19* in brain tissue (C), blood (D), and intestinal tissue (E) of different groups of mice (*n* = 5). (F) Staining of brain tissue of mice in each group under Evans blue tail vein perfusion (F1 displays a composite image of brain tissue, whereas F2 shows images of the same tissue acquired individually), and (G) quantification of EB in brain tissue homogenization using a spectrophotometer (*n* = 3).  ^∗^ *p* < 0.05;  ^∗∗^ *p* < 0.01;  ^∗∗∗^ *p* < 0.001;  ^∗∗∗∗^ *p* < 0.0001; ns: no difference; each symbol represents an individual mouse.

*Streptococcus suis* 2 (*SC19*) is a significant zoonotic bacterial pathogen responsible for severe infections, including meningitis, and is associated with high mortality in both humans and pigs. In our previous studies, strain *SC19* demonstrated significantly higher mortality in mice than strain *P1*/*7* at an equivalent challenge dose. Therefore, the *SC19* strain was selected for this study. As shown in the survival curve, mice infected intraperitoneally with 1.5 × 10^8^ CFU began to die at 12 h postinfection, with 100% mortality occurring around 24 h. In contrast, the low‐dose butyrate group (*SC19* + NaB‐L) showed significantly prolonged survival (*p* < 0.05), and the high‐dose butyrate group (*SC19* + NaB‐H) achieved an 80% survival rate (*p* < 0.01) (Figure 2A). Based on initial optimization of the challenge dose for a murine meningitis model with intra‐abdominal infection, the dose of 1 × 10^8^ CFU was definitively established. This dose elicits clinical neurological symptoms of meningitis without causing mortality. Neurological symptoms were scored on a 4‐point scale, as shown in the color gradient in Figure 2B. *SC19*‐infected mice consistently developed severe symptoms, including head/neck arching and kyphosis. Butyrate intervention markedly reduced these clinical symptoms. Notably, the high‐dose butyrate group was virtually devoid of obvious neurological symptoms, appearing similar to those in the control animals. Consistent with the clinical severity, evaluation of bacterial loads in the blood and brain revealed substantial *SC19* colonization in *SC19*‐group mice. Butyrate intervention resulted in a pronounced reduction in the bacterial burden in these tissues, despite the pathogen remaining detectable (Figure 2C,D). We also quantified bacterial loads in the intestinal tissue (Figure 2E) and confirmed successful *SC19* colonization (Figure S1). Interestingly, the bacterial load trend in the gut paralleled that in the blood and brain. Finally, we evaluated the BBB permeability and integrity to investigate the cause of the neurological symptoms. Imaging of the brain tissue from *SC19*‐group mice visualized extensive dye leakage, confirming BBB disruption. However, administration of butyrate effectively preserved BBB function, maintaining permeability and integrity at levels comparable to those of the Mock group (Figure 2F,G).

### 3.2. Butyrate‐Mediated Protection of BBB Integrity via Enhanced Tight Junctions and Reduced Inflammation

**Figure 3:**
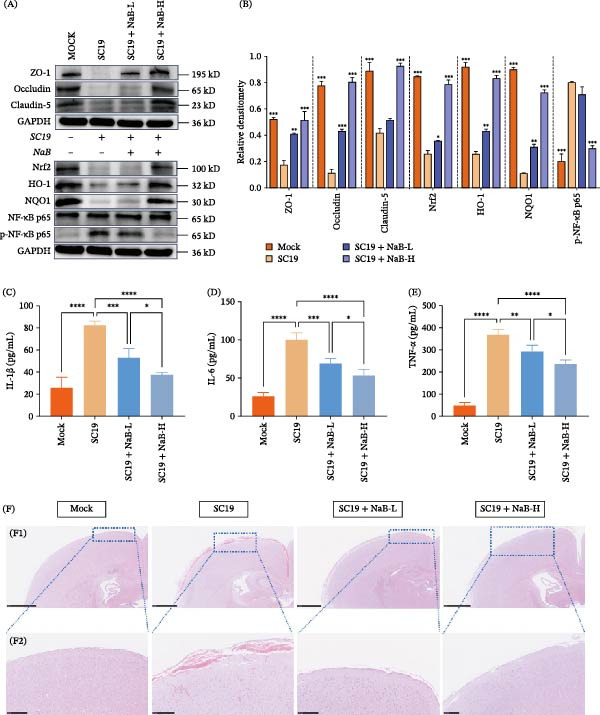
Effects of butyrate on the tight junction, inflammatory response, and histopathology of *SC19*‐infected mice. (A) The protein expression of key tight junction proteins, antioxidant proteins, and their downstream proteins, as well as the inflammatory transcription factor, in the brain tissue of mice. (B) Fusion software was used to quantify and analyze the bands of all proteins (three biological replicates). ELISA evaluation of the secretion levels of inflammatory factors IL‐1β (C), IL‐6 (D), and TNF‐α (E) in the serum of mice in each group (*n* = 5). (F) Histopathological analysis of mouse brain tissue (H&E staining), F1 (1 mm) and F2 (250 μm) (*n* = 5, only representative samples were displayed). ( ^∗^ *p* < 0.05;  ^∗∗^ *p* < 0.01;  ^∗∗∗^ *p* < 0.001;  ^∗∗∗∗^ *p* < 0.0001).

A key mechanism by which *Streptococcus suis* invades the CNS is the disruption of tight junctions, enabling a breach of the BBB and the development of meningitis. To further explore the potential mechanisms behind the series of clinical phenomena caused by butyrate intervention, we initially investigated its effects on tight junction proteins of the BBB. Immunoblotting analysis revealed marked downregulation of key tight junction proteins (ZO‐1, Claudin‐5, and Occludin) in the brain tissues of *SC19*‐group mice compared with the mock group. These results indicate that *SC19* infection significantly impairs tight junction integrity in the mouse brain. Notably, butyrate administration preserved key tight junction proteins (especially Claudin‐5 and Occludin) in mouse brain tissue, maintaining their expression at levels comparable to those in normal physiological conditions. However, this effect was not significant in the low‐dose butyrate group (Figure 3A,B). Given the hypothesized mechanism by which *Streptococcus suis* disrupts the BBB via systemic inflammatory storms and related oxidative stress, we focused our investigation on key mediators of these processes: Nrf2, NF‐κB p65, and their downstream effectors. We confirmed that *SC19* infection significantly elevated NF‐κB p65 phosphorylation and markedly increased the levels of proinflammatory cytokines (IL‐1β, IL‐6, and TNF‐α) in mouse serum. Conversely, the expression of the antioxidant transcription factor Nrf2 was substantially suppressed, consistent with the diminished levels of its downstream effector proteins, HO‐1 and NQO‐1 (Figure 3A,B). Surprisingly, butyrate intervention effectively preserved the physiological expression level of Nrf2, which was associated with elevated expression of its downstream antioxidant proteins HO‐1 and NQO‐1. The expression of p‐NF‐κB p65 and the secretion of inflammatory factors IL‐1β, IL‐6, and TNF‐α were suppressed (Figure 3C–E). Collectively, these results suggest that butyrate protects BBB integrity by preserving tight junction protein expression, likely through antioxidant‐mediated attenuation of the inflammatory response. Finally, H&E staining was performed on fresh brain tissue samples from each group to evaluate the histopathological progression of meningitis. In the *SC19* group, brain tissues exhibited marked meningeal rupture, hemorrhagic congestion, and dense inflammatory infiltration. Notably, perivascular cuffing was present in severe cases, indicating typical parenchymal inflammation. In contrast, butyrate administration markedly alleviated these histopathological signs of meningitis. Brain tissues of the high‐dose butyrate group exhibited no significant microscopic abnormalities (Figure 3F).

### 3.3. Butyrate Stabilizes Intestinal Microbiota Homeostasis Disrupted by *Streptococcus suis SC19*

The confirmed presence of *Streptococcus suis SC19* in the mice’s intestinal tract, primarily in the cecum and colon, prompted us to investigate whether the *SC19* infection induces structural and functional disruption of the gut microbiota. Accordingly, we employed 16S rRNA sequencing to characterize the specific dynamic alterations in the diversity and composition of the gut microbiota in mice. First, we performed alpha‐diversity analysis of the sequencing data to assess microbial richness and diversity across different gut compartments. *SC19* colonization lowered Shannon, Chao1, Sobs, and ACE indices while increasing the Simpson index (Figure 4A), indicating a simultaneous loss of richness and evenness. It is well established that community diversity is positively correlated with the Shannon index and inversely correlated with the Simpson index, while community richness is directly reflected by the Chao and observed species indices. Strikingly, our results indicate that *SC19* infection not only impaired microbial diversity but also reduced the richness, suggesting a marked disruption of the gut’s ecological balance and physiological homeostasis. To dissect the specific effects of butyrate independent of *SC19* infection, a separate NaB‐only control group was utilized. Surprisingly, butyrate significantly enhanced microbial diversity and richness, elevating the Shannon index above that of the control, underscoring its potent positive impact on the gut microbiota.

Figure 4Effects of butyrate on the diversity of microbiota and histopathology in the colonic tissue of *SC19*‐infected mice. (A) Perform alpha diversity analysis of sequencing results to evaluate gut microbiota diversity within each mouse group (the larger the value of Chao, the higher the richness of the community; the higher the Shannon/Simpson values, the higher the diversity of the community). (B) Perform a beta diversity analysis of sequencing results to evaluate differences in gut microbiota composition across groups of mice (*n* = 6). (C) Perform histopathological analysis (H&E staining and PAS staining) on the intestinal tissues of each group of mice to evaluate their intestinal health status, C1 (500 μm) and C2 (250 μm) (*n* = 5, only representative samples were displayed). (D) The depth of colonic crypts in each group of mice was analyzed with Aperio ImageScope software. (E) The average number of goblet cells per 10,000 µm^2^ in the intestines of each group of mice (*n* = 5). ( ^∗^ *p* < 0.05;  ^∗∗^ *p* < 0.01;  ^∗∗∗^ *p* < 0.001;  ^∗∗∗∗^ *p* < 0.0001).

**Figure:**
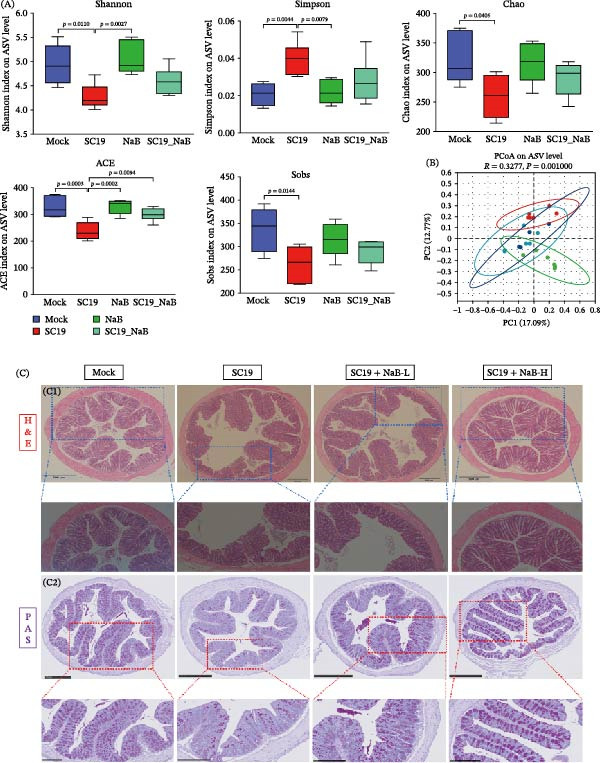

**Figure:**
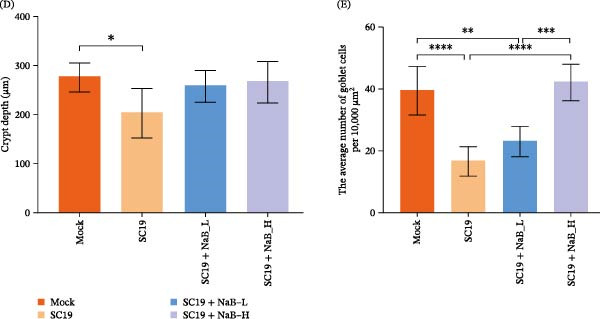

While butyrate administration did not completely reverse the dysbiosis induced by *SC19* infection, it did mitigate it. The microbial community of the *SC19_*NaB group showed overall improvement and enhanced structural stability compared to the *SC19* group, as reflected by a significant increase in the ACE index (Figure 4A). PCoA was performed using Bray‐Curtis distances to visualize compositional similarities and differences among the microbial communities. As illustrated in Figure 4B, the first two principal coordinates (PCo1 and PCo2) explained 17.09% and 12.77% of the observed variation in the community, respectively. Clear separation of the sample clusters among the groups was observed, indicating a statistically significant difference in overall microbial community structure (*p* = 0.001) (Figure 4B). Taken together, these results indicate that *SC19* infection concurrently impairs the diversity and structure of the gut microbiota and that oral butyrate supplementation effectively mitigates this damage.

To complement our investigation, we performed histological analysis of intestinal tissues to assess the impact of *SC19* infection on the gut microenvironment that sustains the microbial community. The impact of intestinal histological changes extends beyond altering the microbial microenvironment to directly affect host systemic physiology through compromised nutrient absorption, highlighting their fundamental role in overall health. H&E staining demonstrated well‐preserved colonic histoarchitecture in both the mock and NaB‐group mice, featuring intact walls, uniformly arranged colonic folds, and clearly visible crypts without notable pathology. This structural integrity indicates a favorable niche for sustaining a healthy microbial community. Unfortunately, *SC19* infection resulted in a substantial erosion of the colonic histoarchitecture. While the overall wall integrity was maintained, the colonic folds were predominantly effaced and disarrayed, and the crypts were indistinct. Such pathological changes would profoundly compromise the colon’s functional capacity for nutrient assimilation and for maintaining microbial populations. Histological evaluation confirmed that the *SC19* + NaB group did not adversely affect colonic integrity after the *SC19* challenge. The colon exhibited preserved structural morphology, with neatly arranged colonic folds and discernible crypts, comparable to those observed in the normal control group (Figure 4C,D). Given that goblet cells protect and lubricate the colon and regulate immunity via mucus secretion, their loss indicates severe barrier damage. We therefore quantified and localized these cells by PAS staining. As shown in Figure 4C, *SC19* infection conspicuously reduced the number and distribution of goblet cells (purple) in the mice colon. Notably, butyrate treatment completely prevented this loss, with goblet cell metrics in *SC19*‐challenged mice remaining at normal levels (Figure 4C,E).

### 3.4. Butyrate Facilitates the Enrichment of Beneficial Microbiota and Augments SCFA Synthesis

The composition and diversity of the gut microbiota are determined by the equilibrium between beneficial and harmful bacteria, which, in turn, regulate host physiological processes through their key metabolic products. Therefore, we performed an in‐depth analysis of the sequencing data, with a focused comparison between the *SC19* and *SC19* + NaB groups, to elucidate the potential mechanisms by which butyrate exerts its beneficial effects on the gut microbiota. Our initial analysis targeted the top 20 genus‐level bacterial species. Following dimensionality reduction and analytical refinement, we compared these taxa to delineate the differences in their abundance and core functional profiles between the two experimental groups. The integrated heatmap revealed that the two most highly enriched genera, [*Eubacterium*]*_siraeum_group* and *Norank_f__*[*Eubacterium*]*_coprostanoligenes_group*, were present at higher abundance in the *SC19* + NaB group compared to the *SC19* group (Figure 5A).

Figure 5Effects of butyrate on the gut microbiome composition, functional classification, and SCFA formation. (A) Heatmap of the top 20 enriched microbial communities at the genus level between the *SC19* group and the *SC19* + NaB group. (B) LEfSe (linear discriminant analysis effect size) bar plot illustrating the differentially abundant genera between the *SC19* group and the *SC19* + NaB group. (C) Wilcoxon rank‐sum test bar plot comparing the relative abundance of genera between groups at the genus level. (D) COG functional classification (predicted gene categories of the gut microbiota). (E) Bar graph depicting the concentration of acetate (F), propionic (G), and butyric acids in serum among the Mock, *SC19*, and *SC19* + NaB groups. (H) Bar graph depicting the concentration of acetate (I), propionic (J), and butyric acids in brain tissue among Mock, *SC19*, and *SC19* + NaB groups (*n* = 6). ( ^∗^ *p* < 0.05;  ^∗∗^ *p* < 0.01;  ^∗∗∗^ *p* < 0.001).

**Figure:**
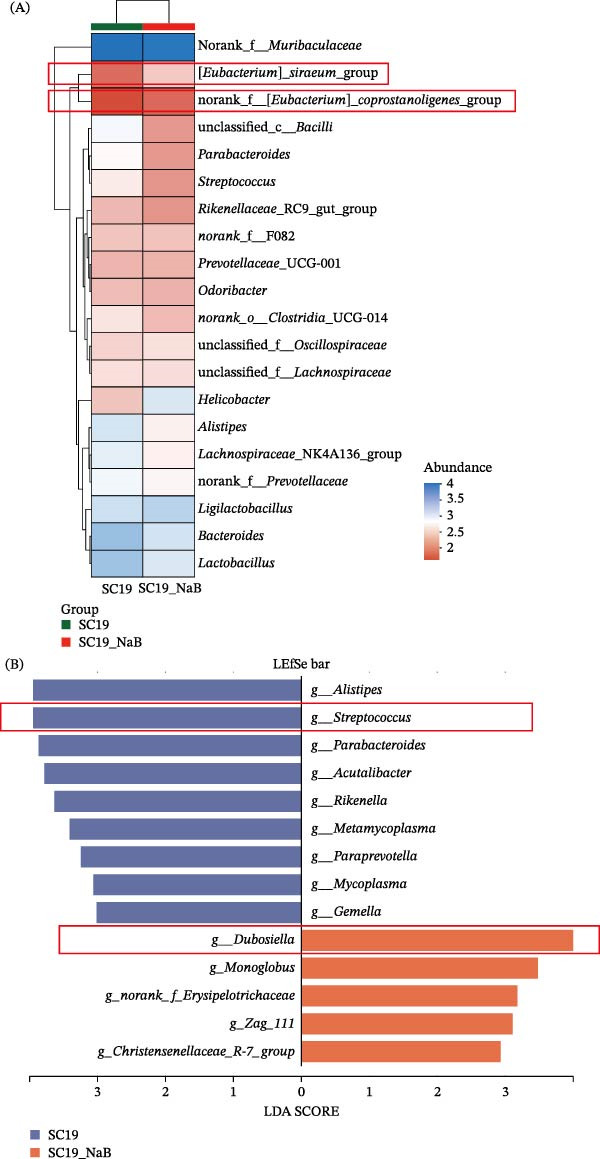

**Figure:**
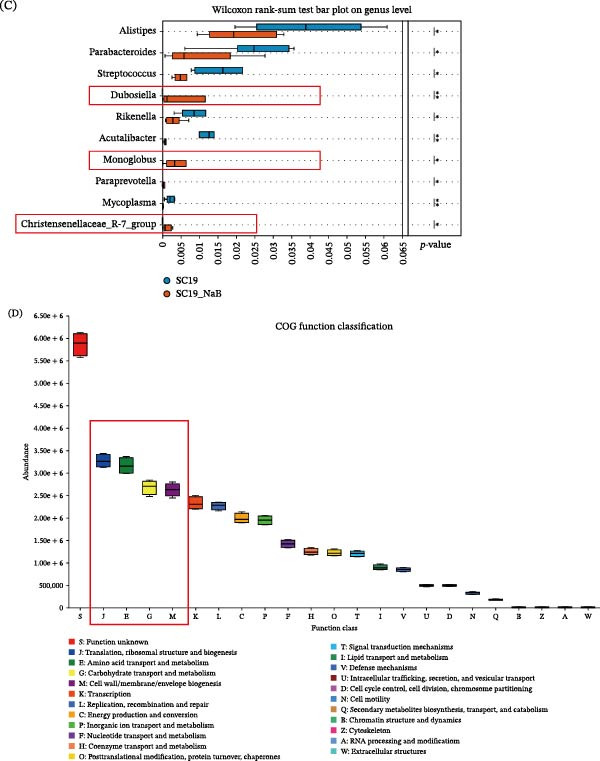

**Figure:**
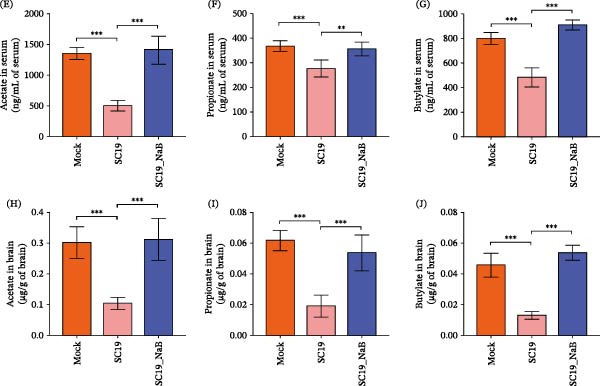

LEfSe analysis was employed to identify key microbial biomarkers distinguishing the two groups. The analysis revealed *g__Streptococcus* (LDA score = 3.95) as the most enriched genus in the *SC19* group, while *g__Dubosiella* (LDA score = 3.99) emerged as the predominant biomarker in the *SC19* + NaB group (Figure 5B). *g__Streptococcus* is a genus of Gram‐positive bacteria classified among the pyogenic cocci. Members of this genus are frequently associated with suppurative infections and inflammatory pathologies, establishing them as significant human pathogens. In this study, the bacterial strain *SC19*, utilized herein as a pathogenic agent, belongs to the genus. Importantly, the genus *g__Dubosiella* is recognized for its role in fermenting dietary fiber to produce SCFAs, including acetate and butyrate. Then, to determine the statistical significance of differences in bacterial genera abundances between the *SC19* + NaB group and the *SC19* group, the Wilcoxon rank‐sum test was performed. We demonstrate that the genera *g__Dubosiella* (*p* < 0.01), *g__Monoglobus* (*p* < 0.05), and *g__Christensenellaceae_R-7_group* (*p* < 0.05) were significantly enriched in the *SC19*_NaB group compared to the *SC19* group (Figure 5C). The enriched genus *g__Monoglobus* (family: *Monoglobaceae*) was identified as a key player in polysaccharide fermentation. Notably, its ability to produce acetate and succinate from complex carbohydrates underscores its potential as a probiotic that degrades dietary fiber and generates SCFAs. The *g__Christensenellaceae_R-7_group* belongs to the *Christensenellaceae* family, which is highly heritable and frequently associated with host health. This bacterial group ferments dietary fiber to produce SCFAs, especially acetate and butyrate. Finally, bioinformatics analysis of the microbial gene sequencing data was performed. The COG functional profile revealed that the dominant microbial communities in the *SC19* + NaB group were predominantly enriched in the following categories: translation, ribosomal structure, and biogenesis; amino acid transport and metabolism; cell wall/membrane/envelope biogenesis; and carbohydrate transport and metabolism (Figure 5D).

To validate the genomic sequencing findings and support the proposed gut‐brain axis hypothesis. We next quantified SCFAs in murine serum and brain tissue by LC‐MS. Consistent with our ratiocination, mice subjected to *SC19* infection exhibited markedly lower concentrations of major SCFAS (acetic, propionic, and butyric acids) in both systemic circulation (serum) and brain tissue relative to normal physiological levels (Figure 5E–G). Compared with the *SC19* group, the *SC19* + NaB group exhibited significantly increased levels of acetic, propionic, and butyric acids in both serum and brain tissue, reaching near‐normal physiological levels (Figure 5H–J). Thus, the intervention effect of butyrate on meningitis and the gut microbiota in mice may be underpinned by its role in sustaining or boosting the availability of SCFAs in the brain and the circulatory system, thereby supporting the involvement of the gut‐brain axis in linking gut microbiota‐derived SCFAs to reduced neuroinflammation.

### 3.5. Butyrate Attenuates Neuroinflammation and Maintains BBB Integrity via the Nrf2/NF‐κB Axis

Our in vivo data demonstrate that butyrate acts via the gut‐brain axis to consequently preserve BBB integrity, thereby countering the pathology of *SC19*‐induced meningitis. To elucidate how butyrate maintains the expression of key tight junction proteins and protects the BBB from damage induced by SC19 infection, we investigated the underlying mechanism in hCMEC/D3. We first investigated whether the beneficial effects of butyrate on the BBB observed in mice could be replicated in vitro using hCMEC/D3 cells, an established human BBB model. Direct *SC19* infection disrupted key tight junction proteins (ZO‐1, Claudin‐5, and Occludin), most notably suppressing the expression of ZO‐1 and Claudin‐5 (Figure 6A,B). Following butyrate administration before the *SC19* challenge, the expression of cellular tight junction proteins was preserved at near‐control levels. In particular, high‐dose butyrate demonstrated enhanced efficacy in maintaining tight‐junction integrity in vitro.

Figure 6Effects of butyrate on BBB integrity via the Nrf2/NF‐κB axis and correlation analysis. (A) The protein expression of key tight junction proteins, antioxidant proteins, and an inflammatory transcription factor in hCMEC/D3 cells. (B) Fusion software was used to quantify and analyze the bands of all proteins (three biological replicates). ELISA evaluation of the secretion levels of inflammatory factors IL‐1 β (C), IL‐6 (D), and TNF‐α (E) in the cell supernatants of each group (*n* = 5). (F) Under Nrf2‐specific inhibitors, the protein expression of key tight junction proteins, antioxidant proteins, and their downstream proteins, as well as the inflammatory transcription factor, was examined in hCMEC/D3 cells. (G) Fusion software was used to quantify and analyze the bands of all proteins.  ^∗^: *SC19* group vs. Mock group and *SC19* + NaB‐H group; ^#^: *SC19* + NaB‐H+Brusatol group vs. Mock group and *SC19* + NaB‐H group. (H) The predicted model of the interaction between butyrate and Nrf2. (I) Spearman’s correlation of clinical symptoms, gut microbiota, and butyrate‐associated molecular pathways between the *SC19* and *SC19* + NaB groups. ( ^∗^ and ^#^ *p* < 0.05;  ^∗∗^ and ^##^ *p* < 0.01; and  ^∗∗∗^ and ^###^ *p* < 0.001;  ^∗∗∗∗^ *p* < 0.0001).

**Figure:**
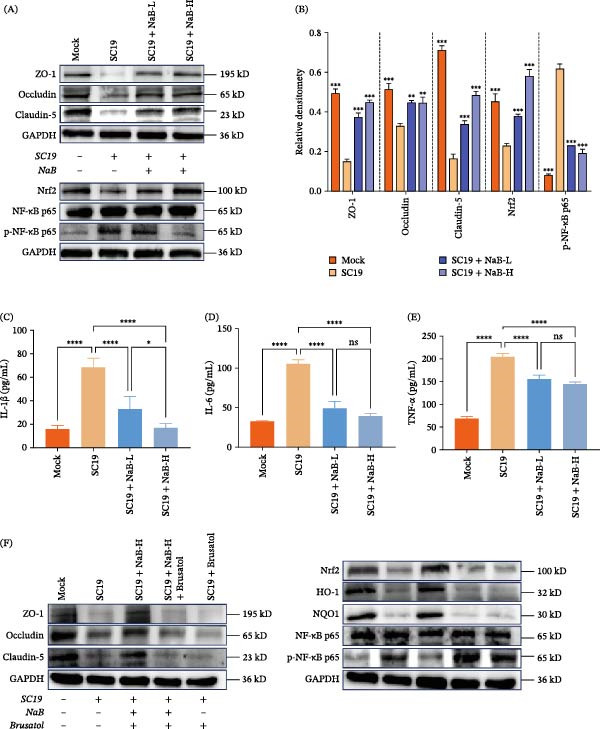

**Figure:**
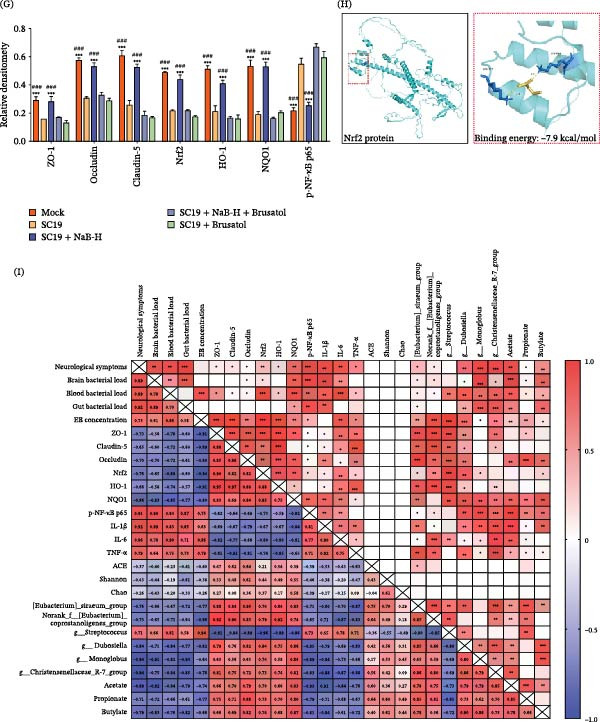

Furthermore, we confirmed that *SC19* infection of hCMEC/D3 cells triggers a pathogenic cascade characterized by a decline in the antioxidant master regulator Nrf2 and concomitant upregulation of the pivotal proinflammatory transcription factor NF‐κB p65, along with its downstream secreted mediators IL‐1β, IL‐6, and TNF‐α (Figure 6C–E). Butyrate intervention reversed this phenotype, sustaining a robust Nrf2 response while curtailing the NF‐κB‐mediated inflammatory cascade. Collectively, these data indicate that the normal expression of tight junction proteins depends on the host’s antioxidant capacity, which appears to restrain inflammatory signaling. The disruption of this balance; therefore, represents a pivotal checkpoint for pathogen invasion.

We hypothesized that butyrate acts upstream of Nrf2, boosts antioxidant defenses, dampens NF‐κB‐dependent inflammation, and in turn preserves BBB integrity. To definitively assess the role of Nrf2, we employed its specific inhibitor, Brusatol (which mediates ubiquitin‐dependent degradation of NRF2 and blocks its de novo synthesis), to determine whether it compromises the beneficial effects of butyrate in *SC19*‐infected hCMEC/D3 cells. We observed that Brusatol significantly suppressed Nrf2 expression in cells without affecting the virulence of *SC19*. In this model, our results delineate the *SC19*‐induced pathogenic cascade: the suppression of the Nrf2 antioxidant pathway (including HO‐1 and NQO‐1), coupled with NF‐κB‐driven inflammation (as indicated by p‐NF‐κB p65), collectively impairs the expression of tight junction proteins ZO‐1, Claudin‐5, and Occludin, leading to BBB failure. Notably, the beneficial effect of butyrate was counteracted in the presence of Brusatol. The degradation of the Nrf2 protein inhibited the activation of its downstream antioxidant pathways (Figure 6F,G). Simultaneously, elevated NF‐κB p65 expression led to a severe reduction in the expression of BBB tight junction proteins, even when administered high‐dose butyrate. Notably, we employed molecular docking techniques supported by large‐scale database models to predict the interaction between butyrate and the Nrf2 protein. The predicted binding mode is shown in Figure 6H; this computational result is exploratory and requires experimental validation. Our data demonstrate that the preservation of BBB integrity by butyrate during *Streptococcus suis* infection is dependent upon both Nrf2 expression and suppression of p‐NF‐κB p65. Finally, Spearman’s correlation analysis of key parameters from the *SC19* and *SC19* + NaB groups was performed to clarify the correlations among butyrate, gut microbiota, SCFAs, clinical meningitis, and the Nrf2/NF‐κB signaling axis in mice. In particular, butyrate content in the serum was significantly positively correlated with the relative abundance of [*Eubacterium*]*_siraeum_group* (*r* = 0.78, *p* < 0.01), *Dubosiella* (*r* = 0.90, *p* < 0.001) and *Monoglobus* (*r* = 0.86, *p* < 0.001), the protein level in the brain of Nrf2 (*r* = 0.74, *p* < 0.05), Occludin (*r* = 0.82, *p* < 0.01), and NQO1 (*r* = 0.87 *p* < 0.01). But it was negatively correlated with the relative abundance of *Streptococcus* (*r* = −0.72, *p* < 0.05), the protein level in the brain of p‐NF‐κB p65 (*r* = −0.81, *p* < 0.01), IL‐1β (*r* = −0.84, *p* < 0.01), the clinical symptoms of meningitis (*r* = −0.79, *p* < 0.01), bacterial load in the brain (*r* = −0.83, *p* < 0.01), and bacterial load in the gut (*r* = −0.82, *p* < 0.01) (Figure 6I). These integrated data corroborate our mechanistic model, revealing a functionally significant interaction between the key metrics and confirming the central role of the gut‐brain axis in this process.

## 4. Discussion

**Figure 7:**
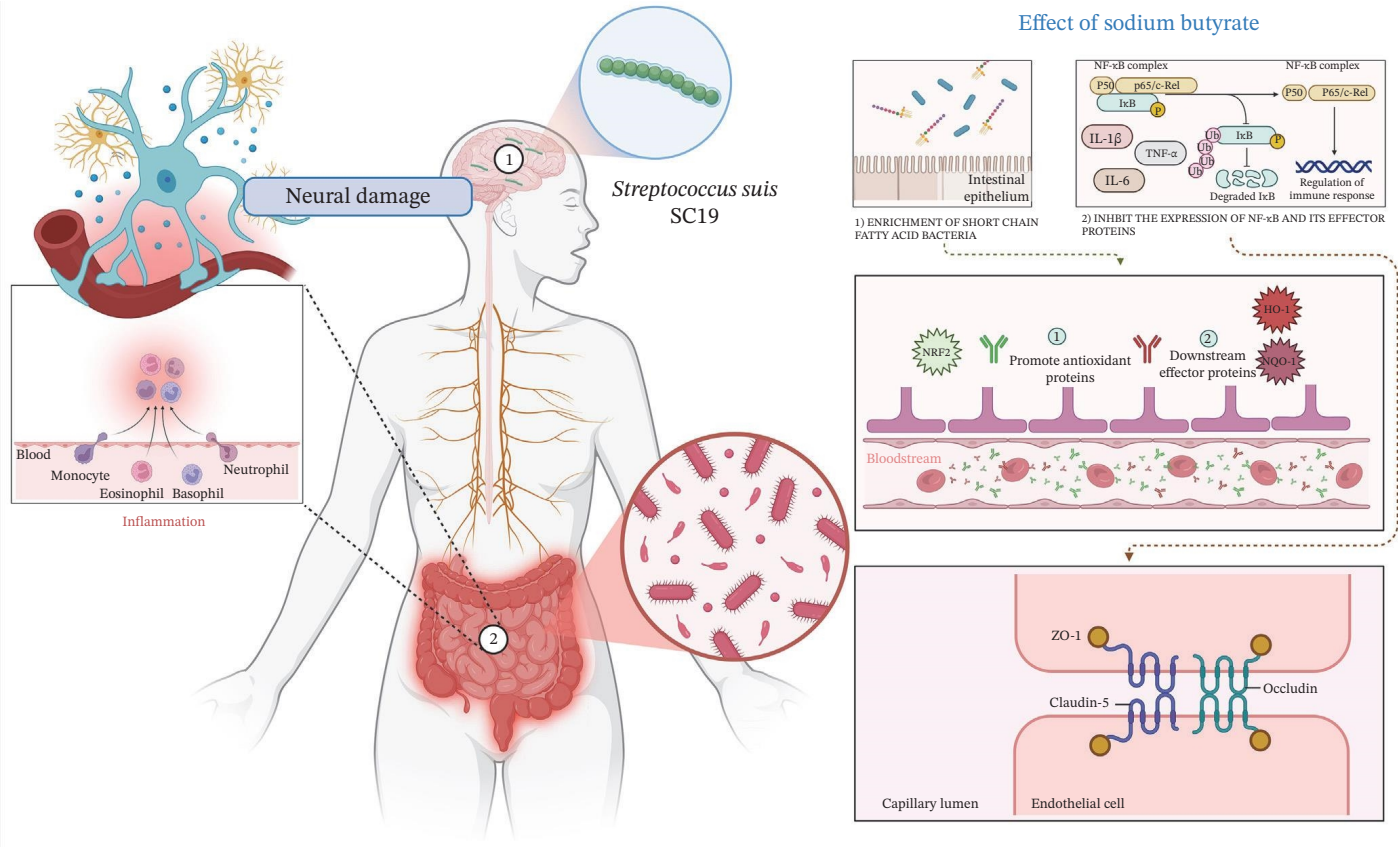
Schematic diagram of the molecular mechanism of butyrate protecting the blood‐brain barrier via the gut‐brain axis.

In this study, we demonstrate that oral administration of butyrate significantly alleviates the pathogenesis of *SC19*‐induced meningitis in mice. The protective effects were evidenced by improved survival, reduced bacterial burden, preserved BBB integrity, and restoration of gut microbiota homeostasis. These changes were associated with elevated SCFA levels and activation of the Nrf2/NF‐κB axis. A critical question arising from these findings is whether the observed benefits are primarily mediated by the gut microbiota via the gut‐brain axis to exert neuroprotective effects (Figure 7).

A hallmark of *SS2*‐induced meningitis is the pathogen’s ability to breach the BBB, which is tightly associated with the disruption of tight junction proteins (ZO‐1, Claudin‐5, and Occludin) and the initiation of a hyperinflammatory cascade [24]. Consequently, infected hosts typically present severe neurological symptoms, and the infection is often characterized by high mortality rates, posing a significant threat to both human and animal health and welfare [25, 26]. Consistent with previous studies, our animal trial data demonstrate that *SC19* infection induces severe neurological symptoms, high mortality, increased BBB permeability, and reduced expression of tight junction proteins in mice. Intriguingly, butyrate intervention dose‐dependently improved survival rates, alleviated clinical symptom severity, and reduced bacterial loads in the blood, brain, and intestinal tissues. Mechanistically, butyrate preserved ZO‐1, Claudin‐5, and Occludin while lowering proinflammatory cytokine output. Together, these effects preserved the BBB structure and, more importantly, functionally limited bacterial entry into the CNS and reduced neurological deficits, as evidenced by lower cerebral bacterial loads, improved neurobehavioral scores, and attenuated histopathological damage in butyrate‐treated mice. These findings align with recent studies highlighting the barrier‐stabilizing properties of butyrate at the BBB. Our work extends those observations from sterile injury models to an infectious context. Hoyles et al. [27] found that propionate, a microbial‐derived metabolite produced in the gut, exerts a beneficial, protective effect on the BBB, a key defensive structure of the brain. Moreover, previous studies have demonstrated that SCFAs can mitigate increased BBB permeability induced by conditions such as sepsis [28], chronic cerebral hypoperfusion [29], and noise exposure [30]. Our findings extend these observations to bacterial meningitis, indicating that the protective effect of butyrate on the BBB is not limited to other disease types but is also applicable to pathogen‐induced barrier disruption.

The gut‐brain axis has emerged as a critical communication network that modulates CNS homeostasis and disease pathogenesis, in which the gut microbiota and its metabolites serve as key messengers [31, 32]. Our 16S rRNA sequencing analysis revealed that *SC19* infection severely disrupted gut microbial homeostasis and altered the community composition. Notably, *SC19* infection depleted SCFA‐producing bacterial taxa, accompanied by reduced levels of acetate, propionate, and butyrate in both serum and brain tissue. Such dysbiosis may exacerbate BBB disruption and neuroinflammation by impairing the production of beneficial metabolites. In contrast, butyrate supplementation restored microbial diversity, enriched SCFA‐producing genera (*Eubacterium siraeum* group, *Dubosiella*, and *Monoglobus*), and elevated systemic and cerebral SCFA concentrations. These findings highlight a bidirectional interplay: *SC19*‐induced gut dysbiosis compromises SCFA production. At the same time, butyrate supplementation enhances the synthesis of beneficial metabolites by remodeling the gut microbiota, thereby establishing a positive cycle that reinforces the BBB integrity. In line with our findings, existing studies have confirmed that genera such as *Eubacterium siraeum* group [33], *Dubosiella* [34], and *Monoglobus* [35] produce SCFAs via distinct metabolic pathways. Importantly, butyrate, as a small, lipid‐soluble metabolite capable of crossing the BBB, possesses a unique advantage in directly transmitting signals from the reconstituted gut microbial ecosystem to the cerebral vasculature, thereby facilitating gut‐brain communication under conditions of peripheral infection [36].

Histopathological analysis of intestinal tissues further substantiates the protective role of butyrate in preserving intestinal barrier integrity and modulating the gut microbiota. *SC19* infection induced pathological alterations, including colonic fold detachment, crypt architectural disorganization, and a reduction in goblet cell numbers, collectively compromising intestinal barrier function and severely disrupting gut microbial homeostasis. Butyrate supplementation reversed these structural abnormalities, maintaining colonic fold‐crypt architecture and goblet cell populations. A functionally intact intestinal barrier, along with a diverse and structurally balanced microbiota, is critical for preventing the systemic dissemination of enteric pathogens and their metabolites, the failure of which exacerbates BBB damage and neuroinflammation [37, 38]. Consequently, butyrate’s ability to stabilize the intestinal barrier likely contributes to its systemic protective effects against *SS2*‐induced meningitis. This dual action, local and systemic, helps explain the magnitude of the clinical benefit observed. These findings align with the gut‐brain axis hypothesis, in which intestinal barrier dysfunction promotes systemic inflammatory responses that ultimately lead to CNS injury [39, 40]. By preserving both intestinal structure and microbial integrity, butyrate restricts the systemic spread of *SC19* and inflammatory mediators, thereby attenuating BBB disruption [41].

The Nrf2/NF‐κB signaling axis serves as a central regulatory hub that balances oxidative stress and inflammation, playing a critical role in maintaining BBB integrity [42, 43]. Our in vitro and in vivo data demonstrate that *SC19* infection suppresses Nrf2 expression and its downstream antioxidant effectors (HO‐1 and NQO‐1) while concurrently activating NF‐κB p65 phosphorylation and promoting the secretion of proinflammatory cytokines (IL‐1β, IL‐6, and TNF‐α). This imbalance between oxidative stress and inflammation directly contributes to tight junction disruption and increased BBB permeability. Butyrate intervention reversed this pathogenic cascade by upregulating the Nrf2/HO‐1/NQO‐1 signaling pathway and inhibiting NF‐κB activation, thereby alleviating oxidative stress and neuroinflammation. Crucially, the protective effects of butyrate were completely abrogated by the specific Nrf2 inhibitor Brusatol, confirming that Nrf2 is indispensable for butyrate‐mediated BBB protection. These findings are consistent with reports showing that HDAC inhibition by butyrate promotes Nrf2 transcriptional activity and drives antioxidant gene expression [44]. Moreover, existing evidence indicates that butyrate suppresses NF‐κB activation by inhibiting IκBα phosphorylation, further supporting its anti‐inflammatory properties [45, 46]. Our study integrates these mechanisms, demonstrating that in SS2‐induced meningitis, butyrate modulation of the Nrf2/NF‐κB axis is a key molecular event linking gut microbial metabolism to BBB integrity.

Spearman’s correlation analysis further substantiated the functional links among butyrate, the gut microbiota, SCFAs, and the Nrf2/NF‐κB axis. Serum butyrate levels were positively correlated with the abundance of SCFA‐producing genera (*Dubosiella*, [*Eubacterium*]*_siraeum_group*, and *Monoglobus*) as well as the expression of Nrf2, Occludin, and NQO1 in the brain. Conversely, butyrate levels were negatively correlated with Streptococcus abundance, cerebral bacterial load, the neuroinflammatory cytokine IL‐1β, NF‐κB p65 phosphorylation, and clinical symptom severity. These correlations robustly support a model in which butyrate, either directly or by enhancing gut microbial SCFA production, activates the Nrf2 antioxidant pathway, suppresses NF‐κB‐driven inflammation, sustains tight junction protein expression, and ultimately attenuates BBB disruption and the pathogenesis of meningitis. This integrated network highlights the gut‐brain axis as a functional conduit through which butyrate exerts its neuroprotective effects.

This study has several limitations that should be acknowledged. First, our study demonstrates the involvement and participation of the gut‐brain axis in butyrate‐mediated protection against *SS2*‐induced meningitis but does not fully and rigorously prove causality. We will employ antibiotic‐depleted recipient mice, fecal microbiota transplantation (FMT), blockade of SCFA receptors (GPR41, GPR43, and GPR109a) [47], and vagotomy to dissect the neural versus humoral pathways. Second, due to the potential off‐target effects of Brusatol, we cannot fully reveal how butyrate exerts and depends on the Nrf2/NF‐κB axis to confer neuroprotection. We have already initiated studies using interfering RNA (siRNA), cellular thermal shift assay (CETSA), and drug affinity‐responsive target stability (DARTS) to more definitively validate the mechanistic pathway. Third, due to lab constraints, our in vitro BBB model is simplified. The hCMEC/D3 cell line lacks pericytes and astrocytes, which are critical for full BBB function. We will establish coculture or organoid BBB models to better recapitulate the neurovascular unit. Fourth, the quantification of SCFAs in the brain tissue requires methodological refinement. Potential issues include residual blood contamination, lack of isotopically labeled internal standards, and inability to distinguish exogenous butyrate from microbially derived SCFAs. We will adopt rigorous protocols including vascular perfusion validation, internal standard calibration, and stable isotope tracing to discriminate administered versus endogenous butyrate. Finally, given the known sex differences in SCFA signaling, we acknowledge the exclusive use of female mice. Both sexes will be included in subsequent studies.

In conclusion, NaB attenuates *SC19*‐induced meningitis by restoring gut microbial balance, activating the Nrf2 pathway, repressing NF‐κB, and thereby maintaining BBB integrity. These findings not only provide deeper insight into the interactions between the gut microbiota and CNS disorders but also suggest a novel preventive strategy against bacterial meningitis associated with BBB disruption. Future work should test nano‐encapsulated butyrate for targeted delivery to the CNS. Parallel efforts should also evaluate combinatorial regimens pairing butyrate with reduced‐dose antibiotics to limit resistance.

## Author Contributions

**Wangyuan Yao:** writing – original draft, validation, methodology, investigation, formal analysis. **Meili Chen**: validation, methodology, investigation, formal analysis. **Huachun Pan**: investigation. **Md. F. Kulyar**: supervision, software, writing – review and editing. **Jialu Zhang**: conceptualization, software, data management. **Hongkai Ren**: data curation, supervision. **Qingqing Luo**: conceptualization, software, methodology. **Hongdou Liu**: formal analysis, data curation, conceptualization. **Qinghua Wang**: supervision, funding acquisition, data curation, conceptualization. **Xuyuan Wang and Rendong Fang**: supervision, software, investigation.

## Funding

This study was supported by the National Natural Science Foundation of China (Grant 32473027), Chongqing Science and Technology Commission (Grant CSTB2025NSCQ‐GPX0495), Fundamental Research Funds for the Central Universities (Grants SWU‐KQ25019 and SWU‐XJPY202305), the Chongqing Modern Agricultural Industry Technology System (Grant CQMAITS202614), and the Co‐construction Project of Fuling Academy of Southwest University (Grant FLYJY202506).

## Disclosure

After using OpenAI, the authors carefully reviewed, revised, and edited the manuscript as needed and take full responsibility for the accuracy, integrity, and content of the publication.

## Ethics Statement

The authors have nothing to report.

## Consent

The authors have nothing to report.

## Conflicts of Interest

The authors declare no conflicts of interest.

## Supporting Information

Additional supporting information can be found online in the Supporting Information section.

## Supporting information


**Supporting Information** Figure S1: Colonization characteristics of *Streptococcus suis SC19* strain injected intraperitoneally in the intestinal tract of mice. (A) Design and validation of specific primers for *Streptococcus suis* type II (F: TTCGTATTAACTTACTTGGCGT, R: TAAATCCCCATATGCCAAATCC, 363 bp). (B) RT‐qPCR for detecting the colonization of *Streptococcus suis SC19* in the intestinal tract of mice. The Cq value is inversely correlated with the SC19 DNA copy number (lower Cq values, higher bacterial load). Comparisons of Cq values between groups reflect relative colonization levels.

## Data Availability

Data are available upon request from the corresponding author.
