# Supplementary material for: Gut Microbiota‐Derived Butyrate Strengthens Blood‐Brain Barrier Integrity and Attenuates Streptococcus suis SC19‐Induced Meningitis: Involvement of the Gut‐Brain Axis
Source: Transbound Emerg Dis. 2026 Aug 18;2026:5502623. doi: 10.1155/tbed/5502623 (PMC13482769; doi:10.1155/tbed/5502623)
Supplement: Supplementary file 1 [file TBED-2026-5502623-s001.docx]

**Supplementary Figure:** Colonization characteristics of *streptococcus suis SC19* strain injected intraperitoneally in the intestinal tract of mice. (A) Design and validation of specific primers for *streptococcus suis* type II (F: TTCGTATTAACTTACTTGGCGT, R: TAAATCCCCATATGCCAAATCC,363bp). (B) RT-qPCR for detecting the colonization of *streptococcus suis SC19* in the intestinal tract of mice. The Cq value is inversely correlated with the *SC19* DNA copy number. (lower Cq values higher bacterial load). Comparisons of Cq values between groups reflect relative colonization levels.).
